# Qualitative application of the diffusion of innovation theory to maternity waiting homes in rural Zambia

**DOI:** 10.1186/s43058-025-00696-y

**Published:** 2025-02-04

**Authors:** Jeanette L. Kaiser, Rachel M. Fiorillo, Taryn Vian, Thandiwe Ngoma, Kayla J. Kuhfeldt, Michelle L. Munro-Kramer, Davidson H. Hamer, Misheck Bwalya, Viviane R. Sakanga, Jody R. Lori, Eden Ahmed Mdluli, Peter C. Rockers, Godfrey Biemba, Nancy A. Scott

**Affiliations:** 1 Department of Global Health, Boston University School of Public Health, Boston, MA USA; 2 School of Nursing and Health Professions, University of San Francisco, San Francisco, CA USA; 3 Department of Research, Right to Care Zambia, Lusaka, Zambia; 4 Department of Health Behavior & Biological Sciences, University of Michigan School of Nursing, Ann Arbor, MI USA; 5 Section of Infectious Diseases, Boston University Chobanian & Avedisian School of Medicine, Boston, MA USA; 6 Center On Emerging Infectious Diseases, Boston University, Boston, MA USA; 7 Office for Global Affairs & PAHO/WHO Collaborating Center, University of Michigan School of Nursing, Ann Arbor, MI USA; 8 Africare, Washington, DC USA; 9 National Health Research Authority, Lusaka, Zambia

**Keywords:** Facility delivery, Obstetric care, Pregnancy and childbirth, Maternal health, Health system strengthening, Sub-Saharan Africa, Qualitative methods

## Abstract

**Background:**

Understanding factors affecting adoption of an innovation is critical to its long-term success. Maternity waiting homes (MWHs) increase access to facility-based delivery in low-resourced settings; yet, quality issues deter utilization of this innovative approach. We sought to understand how attributes that are thought to promote diffusion of innovations (e.g., relative advantage, compatibility, observability, complexity, etc.) affected MWH use after implementation of an improved quality MWH model in rural Zambia compared to standard of care.

**Methods:**

We conducted 158 in-depth interviews (IDIs) with randomly selected rural-living women who had delivered a baby in the prior 12 months. Half lived in catchment areas where new quality MWHs were constructed, half in catchment areas with standard of care (ranging from low quality community structures to no MWH). We applied content analysis to identify themes.

**Results:**

Utilization of MWHs was higher among intervention (65.4%) than control women (42.5%). Respondents in both study arms perceived relative advantages to pregnant women staying at MWHs compared to going directly to health facilities when labor begins. MWH stays allowed for clinical staff to routinely check on and educate women, and address complications immediately. Compatibility of the homes with cultural values and needs depended on implementation. While some women from intervention sites complained about overcrowding, women in control sites more often perceived the lack of cleanliness, amenities, and safety as deterrents to utilization. Women at intervention sites received sensitization about MWHs from a wider range of sources, including traditional leaders. Required preparations needed to stay at MWHs (e.g. delivery supplies, food, and childcare) made adoption complex and may have deterred utilization.

**Conclusions:**

The improved MWH model addressed most community concerns around quality. Having opinion leaders who communicate the relative advantage of MWHs to pregnant women and their social networks may facilitate MWH utilization. The complexity of decisions and resources needed to stay at MWHs remains a critical barrier to use. To facilitate equitable adoption of MWHs among the most vulnerable women, planners should explore how to support women during their delivery preparations and MWH stays, particularly regarding food security and lack of social support for childcare.

**Trial registration:**

clinicaltrials.gov, NCT02620436, Registered 02 December 2015, https://clinicaltrials.gov/study/NCT02620436?term=NCT02620436&rank=1

**Supplementary Information:**

The online version contains supplementary material available at 10.1186/s43058-025-00696-y.

Contributions to the literature

- This is the first application of diffusion of innovation theory to maternity waiting homes as a health system intervention to increase access to skilled obstetric care for remote-living women in low resource settings.
- Emphasizing relative advantage of maternity waiting homes for rapid identification of obstetric complications and their compatibility with current cultural standards and practices could help influence policymakers to adopt maternity waiting homes as essential health infrastructure in rural areas and influence target communities towards use.
- Not recognizing complexity of intervention use is a key barrier to adoption—complexity due to food, resources, and women’s childcare responsibilities must be addressed in future iterations of the intervention.

## Background

Adoption of innovations within a population is slow and not guaranteed, even when the innovation is proven to be effective or useful [1]. According to diffusion of innovation theory, early adopters of innovations inherently differ from later adopters and the speed and breath of adoption depends on attributes of an intervention itself, characteristics of the adopters, and the social, economic, and political context [1–4]. The diffusion of innovation theory put forth by Rogers lists five primary attributes of the intervention that influence adoption: the *relative advantage* the innovation provides over the idea, program, or product it replaces; the *compatibility* of the innovation with the values, experiences, culture, and needs of potential adopters; the *complexity* or difficulty of use; the ability to test before adopting (*trialability*); and the *observability *or tangible results from adoption [1].

Though more frequently used in the fields of technology or management, diffusion of innovation theories, put forth by Rogers [1] and others [2–4], have been applied to medical and public health fields, assessing factors influencing the adoption of health-focused innovations by innovation users [5, 6] (or those performing health behaviors), provider organisations [7, 8] or their members [9], and policy-makers [10]. For example, analyzing the adoption of an innovative intervention for treating chronic back pain, researchers found that the intervention that was perceived to have relative advantages over other possible actions, and that was adaptable and not too complex, was perceived as more acceptable, feasible and appropriate by users [6]. Understanding what aspects of an innovation influence adoption is key to the future implementation, utilization, and sustainability of that innovation.

Maternity waiting homes (MWHs) are one such innovation whose adoption has slowly diffused geographically over the last few decades among governments and organizations seeking to reduce maternal and newborn morbidity and mortality and ensure safe deliveries [11]. MWHs, residential centers built adjacent to health facilities, allow women to reside near an equipped health facility in the weeks prior to their estimated delivery date [11]. MWHs seek to address obstacles women face to delivering with skilled obstetric providers at equipped health facilities, as recommended by the World Health Organization (WHO) to reduce risk of maternal and neonatal morbidity and mortality [11, 12]. Such obstacles include the low density of health services and skilled professionals, long travel times to health services, poor transportation networks, physical barriers such as rivers, and the availability or cost of transportation, all of which can be especially problematic for very remote women in low-and-middle-income countries and when labor begins at night and progresses quickly [13–16].

Prior research from sub-Saharan Africa has found that MWHs are associated with increased odds of facility-based delivery, caesarian section, postnatal attendance, and maternal child health counselling for low resource women; and reduced odds of obstetric complications and poor newborn outcomes [17–24]. However, adoption of the MWHs at the community level, as measured through utilization, has, at times, been limited; the quality of MWHs varies substantially between and within countries and quality is an important and well-documented determinant of MWH utilization [25–28].

Formative research conducted in Zambia elicited community standards for safety, comfort and cultural appropriateness, which key stakeholders and community respondents believed would make MWHs acceptable and increase their utilization [29–31]. The design of an improved MWH model, named the “Core MWH Model” incorporated this feedback in each of its three main pillars: (1) Infrastructure, equipment and supplies to ensure a safe, comfortable, and functional structure; (2) Policies, management, and finances to ensure local oversight and sustainability of the homes; and (3) Linkages and services with the formal health system.

Within a cluster controlled, before and after trial of the impact of the Core MWH Model on facility delivery and MWH utilization amongst remote-living (≥ 10 km from a health facility) rural Zambian women, we collected data from women who had delivered a baby within the prior year at baseline (March 2016), before implementation of the Core MWH Model, and at endline (September 2018) [32]. At baseline, approximately 27% of the cross-sectional sample of recently delivered women had utilized an MWH in the prior year [19]. While this remained the same for control-arm respondents among a repeated cross-sectional sample at endline, it increased to 48% of intervention-arm respondents, indicating a rapid increase in MWH use [19]. This implies a spreading adoption of the MWH intervention among rural Zambian communities with easy access to the improved MWH model but indicates that not all recently delivered women adopted the MWH innovation and utilized it while awaiting their most recent delivery.

To explore the experiences and perceptions of women about access to the improved MWH model, we conducted in-depth interviews as part of the trial’s endline observation. Here we describe factors women perceived to affect their community’s adoption of MWHs, and identify remaining barriers to adoption, guided by the constructs of the diffusion of innovation theory.

## Methods

### Study setting

The Maternity Waiting Homes Alliance was a partnership between the Government of Zambia, two local non-governmental organizations (Right to Care Zambia and Africare), and two American universities (Boston University and the University of Michigan). The Alliance was formed to lessen distance barriers to health facilities in Zambia through the construction of high-quality MWHs in seven rural districts in three provinces.

Over the last two decades, the Government of Zambia has instituted policies to increase access to facility-based delivery, including removing user fees for preventive and primary care services [33, 34]. In addition, between 2012 and 2016, health centers in the seven study districts received renovations and equipment, provider mentorship, and other supply-side interventions to improve the quality of obstetric services through the *Saving Mothers, Giving Life *program [35]. The communities surrounding the health facilities also received messaging from volunteers and traditional leaders to increase demand for facility-based delivery [35]. The baseline observation of the overarching trial found higher than expected rates of facility-based delivery in all districts (81.1%) [19] compared to the national rural average a few years prior (56.3%), indicating the success of efforts to influence delivery location and reduce existing barriers.

Within the before and after impact study, new MWHs meeting the Core MWH Model (explained further below) were constructed adjacent to 20 rural health centers, while an additional 20 matched control sites continued standard of care (also described further below). All rural health centers met eligibility criteria for ability to manage basic emergency obstetric and newborn complications, the details of which were explained further in the published study protocol [32]. At the start of the study, rural health centers within defined geographic areas were matched based on annual delivery volume and government-reported transport time to the nearest referral facility. Half of the sites (under Right to Care Zambia and Boston University) were then randomized to study arm using a simple randomization procedure in Microsoft® Excel; the other half of sites (under Africare and University of Michigan) were assigned to study arm non-randomly due to local government preference for purposeful selection of intervention sites [19, 32]. No substantial differences were found between the randomized and non-randomized groups regarding health facility or catchment area characteristics; within each group, similarities between study arms were even greater [19].

### Intervention design

The Core MWH Model was developed to overcome identified utilization barriers of low MWH quality and lack of amenities, previously identified through formative research in Zambia [29–31] and presented in the literature [25]. At each intervention site, a cement structure was created with pre- and post-natal beds. The sites had latrines, a bathing area, a cooking space, and a veranda for socialization. All clinical care continued to be provided at the health facility according to Ministry of Health guidelines, but health facility staff frequently checked to ensure the MWHs were being properly managed and women attended antenatal care (ANC) visits during their stay. Health staff provided educational talks multiple times per week on a range of topics including newborn care, exclusive breast-feeding, and newborn danger signs. The Core MWH Model was promoted at ANC visits, during outreach activities, and by traditional leadership at community meetings [32]. Women were encouraged to arrive at the MWH approximately two weeks prior to their estimated delivery date, according to WHO and Zambian Ministry of Health guidelines [11].

The 20 control sites continued to practice the standard of care for waiting women in the districts, though it varied substantially in availability and quality. Some control sites had a small community-constructed home which often lacked amenities such as beds or mattresses. Some sites allowed women to sleep in the health facility wards at night and wait on the health facility grounds during the day, while others did not allow women to wait on site [27, 36].

### Description of participants and materials

Data for this analysis were collected between August and September 2018 during the endline observation of the primary study. Data collectors fluent in English and one or more of the four relevant local languages were trained in human subjects’ protection, data collection using Android tablets, and qualitative methods. In all study sites, a household survey was administered to a randomly selected, representative sample of women living more than 10 km from their assigned health facility who had delivered a baby in the past year.

A subsample of the household survey respondents was randomly selected to also participate in a 30-min qualitative in-depth interview (IDI). The previously published IDI instrument incorporated open-ended questions with guided prompts to explore themes on personal and community perceptions of MWH utilization and quality [37]. For the findings presented here, women were asked about their general perceptions of MWHs, reasons why someone in the community might use or not use an MWH, how community members heard about the MWH, and perceptions of improvements needed on their local MWH (if their site had one, which included some control sites where women waiting to deliver slept within the health facility wards).

We conducted IDIs with a random selection of 10% of survey respondents at baseline but met thematic saturation when only 7% were analyzed [38]. Therefore, at the endline observation, we conducted the IDIs with a randomly selected 7% subsample of the survey respondents.

### Analysis

This research is grounded in the Interpretivist paradigm, aiming to understand the meaning behind what respondents said in interviews within the complex cultural, social, and economic contexts and realities in which they exist [39]. Within a Grounded Theory approach to generate hypothesis of differences in perceptions of MWHs between study arms, we utilized the Framework Method for qualitative data management and analysis [39, 40].

First, the IDIs were audio recorded and then translated and transcribed into Microsoft® Word. To ensure accuracy of the translations, transcribers were tested by translating and transcribing two audio recordings prior to being hired. Second, two research staff read and familiarized themselves with the transcripts before coding. We used a mixed inductive/deductive approach [39] to code the transcripts line-by-line in NVivo v.12 (QSR International, Doncaster, Australia). In the deductive approach, the overarching codes were selected a priori using established themes from the interview guide, itself based on prior research and field experience. In the inductive approach, additional sub-codes were generated during coding as themes emerged from the interviews, providing greater clarity of meaning to the overarching codes. Codes were reviewed and merged if there was agreement that two or more codes contained similar content; discrepancies were deliberated on and resolved. Third, the matrix query function in NVivo v.12 (QSR International, Doncaster, Australia) was used to chart and aid the interpretation of themes and patterns in the coded data. Dominant themes (if mentioned by approximately 25% of respondents or more) [39] were summarized and included in the results. Some themes (particularly those around lack of food and childcare responsibilities) that were mentioned by not quite the full quarter of respondents are also presented in the results as they were deemed important by the researchers based on years of anecdotal evidence from fieldwork and prior research. During analysis, we confirmed that results were not driven by any particular study site. Results are presented for the research question on perceptions of MWHs. Quotations illustrating each theme are included in tables [19].We followed the Standards for Reporting Qualitative Research guidelines [41], included as Supplementary File 1.

Demographics, collected during the household survey, were linked to IDI respondents through their unique study ID. Demographics were captured electronically on encrypted tablets using SurveyCTO® Collect software (Dobility, Inc, Cambridge, MA), then cleaned and analyzed in SAS® v9.4 (SAS Institute Inc., Cary, NC, USA). We present demographics for the recently delivered women and their households, and the women’s primary outcomes for delivery location (hospital/health facility or home) and MWH stay.

### Theoretical framework

We interpret the findings of this qualitative study through the lens of the diffusion of innovation theory [1]. We interpret adoption of the Core MWH Model as pregnant women finding the MWH acceptable and utilizing it before labor as opposed to going directly to a health facility from their homes when labor starts or delivering at home. Stages of adoption include awareness of the benefits of the MWH, decision to use or not use the MWH, initial use of the MWH, and intention to use the MWH again in the future, if applicable.

We have applied the five key factors influencing adoption of an innovation to our intervention, shown in Table 1 below. Trialability was assessed during formative research but not for this analysis. We utilized this theoretical framework to compare the perceptions and adoption of women who had ready access to the Core MWH Model (intervention sites) and those who did not (control sites).

**Table 1:** Application of the diffusion of innovation theory constructs to maternity waiting homes

Theory constructs	Application to maternity waiting homes
Relative advantage	Community members believe that using an MWH is better than going to a health facility for delivery when labor starts or delivering at home
Compatibility	MWH is culturally acceptable and meets the needs of the pregnant women staying there
Complexity	How difficult the MWH is to use
Trialability	Extent to which the MWH can be tested before actual use (i.e. through prior stays or touring the new MWHs before needing to stay)
Observability	Extent to which the women are influenced to use the MWH by their peers who have used the MWH and reported back on tangible results. Women may have seen or heard about the MWH while attending health education classes or using services at the health facility

### Data triangulation and corroboration

Qualitative findings were corroborated with both published [19] and unpublished quantitative findings. Additionally, preliminary study findings were provided during community meetings at each intervention site, attended by rural health center staff, representatives from the MWH governance and management structures, and interested community members. These meetings were not specifically targeting participants of the endline data collection, but rather the individuals primarily involved in the implementation and oversight of MWHs. At each meeting, site-specific statistics for utilization and financial sustainability, successes and challenges experienced over the course of implementation, and user and community perceptions, including some of the findings described here, were discussed. Subsequently, study staff held a central dissemination workshop including representatives from each rural health center study site, each district and provincial health office, and the relevant unit at the Ministry of Health. No qualitative themes were modified based on these dissemination meetings. However, they provided greater context and explanation to the subsequent findings.

## Results

### Respondent characteristics

In total, 158 IDIs were conducted among women who delivered in the 12 months prior to data collection (intervention, *n* = 78; control, *n* = 80) (Table 2). Demographic characteristics were similar between intervention- and control-arm respondents. Women were generally in their 20s and 30s and were married or cohabiting. Respondents in both study arms lived a similar distance from their assigned health facility (12–13 km). Households in this study were large (median of 6–7 members) and poor, with no access to electricity and limited use of improved sanitation.

**Table 2:** Demographic characteristics of in-depth interview respondents, by study arm

	**Intervention** ***n* = 78**	**Control** ***n* = 80**
***Respondent characteristics***		
** Woman’s age in years**, median (IQR)	26 (21, 32)	29.5 (23, 35)
** Married/cohabiting**, n (%)	68 (87.2)	71 (88.7)
** Years of education**, mean (SD)	6.1 (3.2)	6.0 (3.4)
** Primigravida**, n (%)	16 (20.5)	10 (12.5)
** Attended four or more ANC visits**, n (%)	60 (75.0)	59 (75.6)
** Age of most recently delivered baby (months)**, mean (SD)	7.0 (3.7)	7.1 (3.6)
***Household characteristics***		
** Household size**, median (IQR)	6 (5, 8)	7 (5, 8)
** Dependency ratio**^**a**^, mean (SD)	1.5 (1, 2)	1.5 (1, 2)
** Travel distance from home village to health facility (km)**, median (IQR)	12.1 (11, 15)	13.2 (11, 16)
** Electricity**, n (%)	1 (1.3)	1 (1.3)
** Improved sanitation**^**b**^, n (%)	12 (15.4)	21 (26.3)
***Study outcomes***		
** Delivery location**, n (%)		
* Hospital/Health facility*	75 (96.1)	72 (90.0)
* Home/Other home*	2 (2.6)	7 (8.7)
**Heard of an MWH**, n (%)	72 (92.3)	73 (91.2)
**Utilized MWH during most recent delivery**, n (%)	51 (65.4)	34 (42.5)

*Abbreviations: IQR* interquartile range, *SD* standard deviation, *ANC* antenatal care, *MWH* maternity waiting home

^a^Dependency Ratio = (children < 16 years old + adults > 65 years old)/adults > 16 years old

^b^Improved sanitation includes flush or pour toilets; ventilated, improved pit latrines; and pit latrines with slab

As expected, more respondents from intervention sites reported having used an MWH (65.4%) compared to control sites (42.5%). A smaller difference was found for delivery at a health facility (intervention: 96.1%; control: 90.0%). Both study arms showed high knowledge of MWHs (intervention: 92.3%; control: 91.2%).

### Relative advantage of MWHs

Respondents in both study arms found utilizing the MWHs advantageous compared to going directly to the health facility for delivery or delivering at home (Table 3). Respondents highly valued having health staff nearby in case they experienced any pregnancy complications and liked that health staff checked in on the waiting women. Additionally, respondents in both study arms liked the convenience of going from the MWH to the health facility once labor started, stating that the distance is significantly shorter than if they traveled directly from home (Table 4, quotes a and b).

**Table 3:** Qualitative themes for each diffusion of innovation theory constructas expressed by in-depth interview respondents, by study arm

	**Intervention-arm respondents**	**Control-arm respondents**
**Relative advantage**	• **Attended by health staff**: staff checked on, taught and encouraged women; staff can help with complications immediately; health facility linked with MWH • **Near health facility**: can go quickly to the facility from MWH to deliver once labor starts; helpful for those who live far	• **Attended by health staff**: staff can help with complications immediately; staff checked on women • **Near health facility**: can go quickly to the facility from MWH to deliver once labor starts; avoid delivering on the way; helpful for those who live far
**Compatibility**	• **MWH quality**: built well; comfortable; had needed amenities and facilities • **Safety**: felt safe, like home; no theft; protected from diseases such as malaria and those linked to poor sanitation • **MWH managed well**: kept clean; taken care of by health facility staff and MWH worker • **MWH too small**: MWH overcrowded, some women end up using the old MWH; not enough beds, some sleep on the extra mattresses	• **MWH quality**: women “stay well”; a place to sleep and cook; easier to stay if prepared • **Poor quality of MWH**: not comfortable; facilities in bad condition and need repair; no beds or mattresses; dirty; no cooking area; no water or electricity • **Safety**: felt safe; near trained health staff; protected from diseases (not specified) • **Lack of safety**: shared space with other people waiting for sick relatives; need wall or fence; no door • **MWH managed well**: health staff managed the MWH; kept clean • **MWH too small**: overcrowded; pregnant women mixed with other people waiting for sick relatives; no place to put belongings
**Complexity**	• **Lack of preparation**: no money or ran out of money to use during stay for food and other items; did not buy baby clothes or required delivery supplies • **Not enough food**: ran out of food and cannot go back home to get more; no money to buy food during stay • **Distance & transportation**: far distance from home to MWH; lack transport	• **Lack of preparation**: no money or ran out of money to use during stay for food and supplies; did not buy required delivery supplies or baby clothes • **Not enough food**: not enough food to take to MWH • **Children and husband at home**: family relies on woman at home; no one to stay with children at home
**Observability**	• **Sensitization about MWH:** received messaging about the MWHs from health facility staff, volunteers, and traditional leaders; also heard benefits from other community members and from radio messaging	• **Sensitization about MWH:** received messaging about the MWHs from health facility staff and volunteers

**Table 4:** Illustrative quotes for emerging themes expressed by in-depth interview respondents, by study arm

	**Intervention-arm respondents**	**Control-arm respondents**
Relative advantage	**a.** “The MWH is near the clinic. You can walk from the MWH to the clinic and deliver; it is a shorter distance. The village is far and they built that MWH to help us find it easy to utilize the service without spending much on transport and for those with no means, they also assist them while at the MWH.” – Woman, Kalomo District	**b.** “[Women] use them [MWHs], because they want to be closer to the facility, rather than going when they are due. They want to avoid delivering on the way and in the village. Even after delivery they will stay there [at the MWH] for observations, maybe for three days or more.” – Woman, Mansa District
Compatibility	**c.** “The place is clean, well furnished with mattresses, mosquitoes and other requisites including beds and beddings. These are helpful because not every woman is able to afford them. They also have water, electricity, and toilets.” – Woman, Mansa District	**d.** “We cook for ourselves so that we stay well at the MWH. We cook at the MWH with no problems.” – Woman, Choma District **e.** “Others don’t go because they fear that they will be sleeping on the floor and experience a lot of discomfort including body pains. There are no beds, sometimes water is a problem or there is no electricity.” – Woman, Choma District
Complexity	**f.**”What makes it hard sometimes is the fact that women are unprepared in terms of requirements for when the baby arrives. Sometimes they rely on old clothes or the nappies are worn out or stained and they feel embarrassed to take them to the MWH.” – Woman, Choma District **g.** “Most women complain about food, because we go very early and spend more on food and other things, transport also. It happens that you go rushing [to the MWH] without any money. But, otherwise, it is okay.” – Woman, Chembe District	**h.** “The children are home alone and rely on you to provide for them, so it’s difficult. Personally, I would not love to go there [the MWH], because I have no one to care for my children.” – Woman, Chembe District
Observability	**i.** “The nurses do teach [and] raise awareness about the MWH. The community volunteers as well do a lot of encouraging [even] more than the nurses and also our spouses and village headmen.” – Woman, Pemba District **j.** “During community meetings or gatherings organized by village headmen, they make announcements about the availability of these MWHs. They also encourage women to utilize these MWHs during the same meetings. – Woman, Kalomo District	**k.** “The health facility staffs do give out the information about the MWH during health education when women go for ANC. The health facility staff tell us that when your pregnancy is eight months, you should come and stay close to the clinic.” – Woman, Lundazi District

### Compatibility of MWHs

Respondents generally found the MWHs compatible with their cultural values and needs, perceiving the quality of the MWHs to be good overall. Respondents from control sites reported “staying well” at the MWH because they had a place to cook and sleep, even though there may not be any beds or mattresses, and they did not have to do any chores (Table 4, quotes d and e). However, they emphasized that being prepared by bringing money and supplies (e.g. food, cooking oil, toiletries) was key – it was easier to use the MWH if one was prepared. Compared to the respondents from control sites, respondents from intervention sites were more emphatic in their responses, expressing excitement about staying at the MWH due to its good quality (well-built and clean) and available amenities, such as clean latrines and a place to bathe (Table 4, quote c). They reported finding everything they needed at the MWH, such as beds, mattresses, blankets, and cooking utensils, and appreciated not needing to bring much from home other than food to cook. Respondents from both study arms also found the MWHs to be safe and well managed.

While perceptions of quality were generally positive, compatibility concerns were raised by respondents from control sites and a few from intervention sites. Many respondents from control sites perceived the MWH as poor quality due to the lack of beds and mattresses, forcing pregnant women to sleep uncomfortably on the concrete floor. Respondents from control sites also cited concerns about the MWH being dirty, having no separate cooking area, and no access to water and electricity (Table 4, quote i). Furthermore, respondents from control sites perceived the MWH as needing repair, with the structure and latrines in bad condition. Some respondents from control sites found safety at the MWHs to be a concern due to lack of a safe place to put their belongings, no fence or door, and sharing the space with many other people, including relatives of sick patients and not just other waiting pregnant women. A few respondents from intervention sites found the MWH to be too small, but they did not mention other compatibility concerns.

### Complexity of MWHs

Complexity of utilizing the MWH was discussed as a main deterrent by both study arms. Staying at an MWH required advanced planning and gathering of supplies (particularly food). Not having enough money to buy food or other items or not coming prepared with the required items for a facility-based delivery deterred MWH use (Table 4, quotes f and g). Respondents from both study arms reported that it was not feasible to go home for more food if they ran out. They either had to purchase more food on site or rely on a family member to bring some.

Respondents from control sites also discussed time away from home as a major deterrent to MWH use, because their children and husbands remained at home while they were awaiting delivery. They discussed the need to identify someone to care for the children or other relatives when they went to the MWH, and the challenge of splitting food between themselves and the needs of their home (Table 4, quote h). This was not a dominant theme amongst respondents from intervention sites.

Respondents from intervention sites also cited the far distance from their home to the MWH and lack of transportation as challenges. However, respondents from intervention sites acknowledged that they had more time to plan transportation or walk to the MWH compared to urgently seeking transportation after labor has begun. This was not widely discussed amongst respondents from control sites.

### Observability of MWHs

MWHs were on the grounds of health facilities and generally accessible to respondents, or at least viewable. Respondents in both study arms discussed receiving messaging about the MWHs and its benefits from health facility staff, particularly during ANC visits, and from community-based health volunteers (Table 4, quotes i and k). Many respondents from intervention sites also discussed how traditional leaders (chiefs and village headmen) had promoted the MWHs during community meetings (Table 4, quote j). Some respondents from intervention sites heard about the MWH from other community members who stayed there, or on the radio. These additional sources were not widely discussed by respondents from control sites.

### MWH improvements needed

When asked how the MWH could be improved, respondents in both study arms recommended expanding the MWH (Table 5). They explained that the MWH was often overcrowded, so there was a need to expand the sleeping and cooking areas to accommodate more women (Table 5, quotes a and b). Additionally, respondents from control sites emphasized the need for beds, along with providing blankets and mosquito nets so that they will not have to carry these items from home. A few respondents from control sites suggested the need for better management of the MWH, such as by having the community and waiting women keep the MWH clean and the need to implement rules for women to follow to prevent the MWH from getting damaged.

**Table 5:** Qualitative themes and illustrative quotes for improvements needed at maternity waiting homes by core maternity waiting home model pillars, by study arm

	**Intervention-arm respondents**	**Control-arm respondents**
**Structure/ Amenities**	• **Need to expand**: overcrowded; need bigger sleeping and cooking areas to accommodate waiting women	• **Need to renovate and expand**: space too small, overcrowded; need rooms and separate cooking area; build another MWH • **Need beds & bedding**: no or not enough beds, mattresses, blankets, and mosquito nets
**Management**	No themes emerged	• **Need to clean space: **MWH not clean, need to keep MWH clean • **Need rules:** implement rules to follow so the MWH doesn’t get damaged or vandalized
**Linkages with health system**	No themes emerged	No themes emerged
**Illustrative Quotes**	**a. **“The big thing that people are talking about is that they want the MWH to be expanded. That place is small and during the dry season, there is not enough space for cooking and sleeping.” – Woman, Choma District	**b. **“It can be improved by extending it [the MWH] with toilets so that it can be divided from where we sleep and where we cook from.” – Woman, Mansa District

## Discussion

Though MWHs were well known in both intervention and control communities, utilization (i.e. adoption) of the homes was higher in communities with improved homes (65%) compared with unimproved homes (43%) [19]. We explored perceptions around adoption in communities with and without improved MWHs within the framework of the diffusion of innovation theory, which has not previously been applied to MWHs as a health system intervention. While respondents expressed similar motivations toward adoption, finding similar relative advantages of the innovation, and receiving similar sensitization about the homes, improved homes were more compatible with respondents’ cultural values and needs. The complexity of utilizing the homes remained a challenge in both study arms.

MWHs provide an important relative advantage above the benefits of facility delivery alone, recognized by respondents in this study and consistent with findings from previous research in rural Zambia [42]. Health facility staff in these rural Zambian districts similarly highlighted the benefits of monitoring women more closely during their stay, allowing health facility staff to provide more timely and comprehensive obstetric care during the final stage of pregnancy [43]. A recent realist review of MWH literature in low- and middle-income countries found similar results: if women perceive positive benefits from MWH use, they are more likely to adopt the intervention [28]. Governments or implementers should communicate the relative advantages of MWHs when seeking to increase their utilization.

Health facility staff, community-based volunteers, and traditional leaders are well placed within this context to sensitize communities and fill the critical role of opinion leaders and champions for adoption previous diffusion theories have highlighted [3]. Furthermore, reinforcement of messages from multiple sources may have a compound influence on utilization [44]. In addition to pregnant women, communicating the relative advantage of MWHs among pregnant women’s social networks and wider communities is important, as these individuals often influence women’s decision to utilize MWHs and deliver at health facilities [45]. Additionally, positive perceptions of MWHs within wider communities have been shown to increase community ownership of the homes, contributions to their operations, oversight, and sustainability of homes, and support to women in their decision to utilize the homes [28, 46].

The Core MWH Model was successfully responsive to community input; findings for the compatibility construct were most positive among respondents in communities with improved homes. Poor MWH quality was the main use deterrent the Core MWH Model aimed to address by meeting the expressed cultural and physical needs of these communities [32]. Expectedly, in control sites with no MWH, where women wait in the wards of the health facility, or with an unchanged community-constructed structure, the poor built environment and lack of amenities remained a substantial deterrent to use. The findings by respondents from control sites are consistent with those expressed during formative research [30, 31] and more widely within the literature [25, 28]. However, overcrowding remained a concern among intervention and control women alike, highlighting the difficulty of appropriately planning for MWH bed capacity even for improved homes, as discussed by Vian et al. (2022) [47]. Additionally, concern shown by respondents from control sites for safety, lack of cleanliness, and lack of maintenance highlights the need for MWH oversight, accountable individuals, and clear policies at MWHs, which have been called for in previous literature [30, 31, 48, 49]. Inclusion of formalized governance and management structures [50], such as those set out within the Core MWH Model, are important for MWH implementation and sustainability.

The complexity of the intervention for users remained a meaningful barrier for respondents in both study arms. Food insecurity was particularly highlighted, corroborating findings from other MWH studies [30, 51, 52]. Overcoming food insecurity during the likely multi-week MWH stays (recommended starting 14 days prior to estimated delivery date) could be an important strategy to increasing utilization and is a critical concern for equitable access. A recent cross-sectional household survey in Ethiopia found that women were four times as likely to utilize an MWH if it provided food [53]. Innovative ways to address food insecurity in this context are needed as it remains one of the key barriers to innovation adoption after quality concerns are addressed. Previous authors have suggested novel solutions such as farming local-acceptable indigenous insects as sources of protein for women staying at MWHs [54, 55].

Though the complexity and resources needed for delivery preparations were raised by respondents as barriers to MWH adoption, quantitative data for the larger trial showed no significant differences in delivery expenditures between MWH users and non-users [56]. The primary cost driver was a new baby blanket, which is more of a cultural expectation than a healthcare system requirement [56, 57]. Delivery preparations are required by the health system (i.e., bringing delivery supplies) or culturally expected (i.e., baby clothes and blanket) regardless of whether a woman utilized a MWH while awaiting delivery. This has been shown by studies in rural Zambia [30, 31, 42, 57, 58] as well as in other contexts, including Ghana [59] and Haiti [60]. Birth preparedness and savings counseling during ANC visits could help women save in preparation for both MWH stays and facility-based deliveries [61, 62].

Corroborating previous literature from rural Zambia [45], respondents from control sites raised concerns around childcare and competing responsibilities hindering their ability to utilize MWHs. Previous studies in Ethiopia found similar results: women with young children were more likely to use MWHs if they had childcare support [53, 63]. Similarly, a broader review of the MWH literature found social support for completion of household commitments in their absence, including childcare, cooking, and cleaning, facilitated MWH utilization [28]. Concerns around childcare and household responsibilities were rarely mentioned by the respondents from intervention sites in our study; it is not clear how respondents in the intervention sites overcame this concern or why these respondents may have had greater social support. We hypothesize that greater communication about MWH benefits from opinion leaders in the community, particularly traditional leaders, may have fostered increased understanding of the relative advantages of MWH stays within the woman’s social network, and increased their willingness to offer support.

Similarly, while far distance from home and lack of transportation were raised as key barriers to use of MWHs among intervention women, these were not raised by many control women. The authors hypothesize that for control women, lack of childcare or support for household commitments may have superseded challenges related to distance and transportation, leaving less time during the interviews for discussion of these other challenges. However, these concepts are interlinked: far distances make staying at an MWH more complex, necessitating transportation to get to an MWH, requiring the splitting of food between the household and the waiting women, and making waiting women unable to easily return home to assist with household chores and provide childcare. While few prior studies have described distance alone as a factor making MWH utilization more complex, transportation costs and lack of transportation options have received more attention in the MWH literature [25, 64]. Yet, MWHs are meant to mitigate the difficulties of distance and transportation costs by allowing transport to be planned, alleviating the need to utilize rapid and more expensive methods of transportation at inconvenient times, such as when labor begins at night [57].

While, this study focused on the characteristics of the MWH innovation for its adoption among the target population, previous authors have theorized that characteristics of the adopters and the socio-political and economic contexts are also critical to the diffusion of any innovation [3, 4]. Previously identified individual and household characteristics associated with MWH use included perceptions of and/or previous experiences with the health facility, status as a young or primiparous woman, financial means for a MWH stay, and positive views of MWHs by household decision-makers or the woman being a joint decision-maker [28]. Additionally, contextual factors include the quality of care and financial and human resources of the health system, how well the health system accommodates cultural beliefs around pregnancy and delivery, and widespread poverty [28].

Though our findings were similar to those of other studies, use of the diffusion of innovation theory allowed us to categorize our findings in a useful manner, that may be easier to communicate to future implementers. It proved useful to conceptualize MWHs as an innovation whose adoption can diffuse throughout a population as early adopters share their experiences and more influential community leaders highlight the MWHs’ relative advantages and compatibility, leading to higher adoption rates. We recommend other authors utilize this theory for similar health system interventions. Additional research could explore more closely how adoption of health interventions diffuses throughout a community in real time and the individual, social, and contextual influences on early, late, and non-adopters.

### Study strengths and limitations

This study was conducted in 40 rural health center sites across four rural districts in Zambia. The results represent a wide swath of women’s perspectives across rural Zambia. However, this study was not implemented in a silo, and spillover of messaging, community perceptions, and uptake of the intervention occurred. Previous quantitative findings from this impact study indicate that MWH awareness increased over time, regardless of study arm [19]. Additionally, the Government of Zambia constructed an MWH similar in quality to the Core MWH Model at one control site during the implementation period, though this facility lacked a community-led governance and management structure. The themes presented were identified in multiple study sites, suggesting the factors influencing MWH utilization were important in multiple communities.

The 20 MWHs implemented under this study operated for a minimum of 13 months at the time of data collection, which may not have been enough time to see diffusion of the innovation and major shifts in community perceptions. While adoption of MWHs may be slow, it is clear some shifts in community perceptions have occurred in the communities that experienced the introduction of quality MWHs, as 50% more intervention women stayed at an MWH compared to those in the control group. Additional shifts may be seen with longer operation of the homes.

This analysis was novel as the first known application of the diffusion of innovation theory to MWHs. The theory’s constructs are broad, which allowed for flexibility when applying the theory to MWHs. However, while the constructs of relative advantage, compatibility, and complexity were easily applied to MWHs, trialability and observability were not as straight forward. Though, hypothetically possible, trialability (extent to which the MWH can be tested before actual use) would not have been feasible in many facilities that experienced frequent overcrowding. Respondents were not asked whether they toured or tried the MWHs before use. While the construct of observability was originally described as the extent to which outcomes can be seen by potential adopters [2], we adapted the definition for our analysis as peer influence by other MWH users or sensitization through trusted sources of information. Tangible results from use of the MWH are not easily observed by outsiders unlike other types of innovations. Rather potential adopters can primarily only rely on reports from other users or influential individuals in their networks.

## Conclusions

To increase equitable access to MWHs and adoption among the most vulnerable rural women experiencing food insecurity and lack of social support, future interventions and policies must address the complexity of use. In addition to addressing compatibility concerns and communicating the relative advantages of MWHs, future MWH interventions should focus on how to better support women during their stay to address and use opinion leaders to sensitize wider communities.

## Supplementary Information

Supplementary Material 1.

## Data Availability

Qualitative data are available in a public, open access repository. https://hdl.handle.net/2144/44014

## Abbreviations

ANC: Antenatal care
IDI: In-depth interview
MWH: Maternity waiting home
WHO: World Health Organization
